# Lived Experiences of Fragile X Syndrome Caregivers: A Scoping Review of Qualitative Studies

**DOI:** 10.3389/fneur.2020.00128

**Published:** 2020-02-27

**Authors:** Karen Kengne Kamga, Jantina De Vries, Seraphin Nguefack, Syntia Nchangwi Munung, Ambroise Wonkam

**Affiliations:** ^1^Division of Human Genetics, Department of Pathology, University of Cape Town, Cape Town, South Africa; ^2^Department of Medicine, Faculty of Health Sciences, University of Cape Town, Cape Town, South Africa; ^3^Department of Pediatrics, Faculty of Medicine and Biomedical Sciences, University of Yaoundé 1, Yaoundé, Cameroon; ^4^Institute of Infectious Disease and Molecular Medicine (IDM), Faculty of Health Sciences, University of Cape Town, Cape Town, South Africa

**Keywords:** fragile X syndrome, lived experience, care givers, scoping review, qualitative research

## Abstract

Fragile X Syndrome (FXS) is the most common x-linked monogenic cause of Intellectual Disability (ID) and Autism Spectrum Disorder (ASD). Taking care of children with ID is challenging and overwhelming due to the multiple facets of caregiving. This scoping review aimed at summarizing the qualitative literature on the experiences of families living with FXS, identify key themes and determine the gaps in the extant literature. We conducted a literature search in May 2019 using four databases; PubMed, Web of Science, African-Wide-Information, and Scopus. The keywords used in our search strategy were associated with caregivers, lived experiences, FXS, and qualitative research. All English language articles with full-text reporting were included. Studies associated with other neurodevelopmental conditions and quantitative studies were excluded. We identified 12 out of 203 articles that described the lived experiences of families with FXS. Most articles originated from the United States of America and mothers were the main caregivers. We summarized our findings into four major themes which are; grief experiences, challenges of living with FXS, coping mechanisms and the need to plan for future outcomes. This scoping review highlights the scarcity of qualitative FXS literature in the African population and frustrations endured by families with FXS due to the low knowledge of FXS by healthcare workers. More research is needed to evaluate the impact of living with FXS in males and fathers.

## Introduction

Fragile X syndrome (FXS) is the most common inherited single-gene condition that causes a range of developmental problems, including learning disabilities and cognitive impairment and affects about twice as many males as females (1). FXS is caused by a mutation of the Fragile X mental retardation 1 (*FMR1*) gene, which results from an expansion of the CGG(Cytosine-Guanine-Guanine) repeats at the 5′ UTR of this gene. Fragile X Mental Retardation Protein (FMRP), the translation product of the *FMR1* gene, is a regulation factor that controls most of the proteins important for synaptic maturation and plasticity (2). A mutation in the *FMR1* gene switches off the production of the FMRP, leading to the weakening of synapses in the brain. This eventually leads to impaired brain development and the physical impairments associated with FXS (3).

In Europe, America, Asia and Australia where molecular diagnostic procedures are well-developed, neonatal units for early screening for genetic diseases like FXS are fully functional and FXS is usually diagnosed early. This could account for the perceived high prevalence in these regions (4, 5). However, not many hospitals and institutions in Africa can perform genetic testing for *FMR1* mutations. Therefore, many African FXS patients may have gone undiagnosed due to the reliance on clinical signs and symptoms (6, 7) and no referral of patients to specialized centers (6). The low uptake of genetic test in African settings could also be due to scarce medical genetics specialist in many African countries. For example, in South Africa in 2013, there were reported to be 11 registered medical geneticists, 42 medical genetic scientists and technologists, and 10 genetic counselors servicing a population of 51 million (8). In other African countries, “medical genetics” services are far fewer and are still developing (9). Consequently, there have been fewer African FXS-related studies published, that can also contribute to the lack of awareness of FXS, insufficient diagnostic tools and possibly cultural believes surrounding mental disorders (10).

Once diagnosed, the medical management of FXS requires family support which is demanding, overwhelming and can lead to some mental health problems to the caregiver (11). Some scholars examined factors which account for parental adaptation to a child's disability. They highlighted that coping with a disability is a multifaceted event which can be experienced in different ways. An alteration in the behavior and sleep patterns of FXS children may account for the maladaptation of parents (12, 13). Hence, this scoping review will map the available evidence of the experiences of families, caregivers and patients living with FXS. We aim at summarizing current lived experiences of FXS caregivers, synthesize key findings, and highlight gaps and limitations in the extant literature.

## Methodology

The scoping review method is a rigorous approach to literature review, systematically identifying key concepts, theories, evidence, and research gaps (14, 15). This scoping review covers empirical qualitative studies reporting on the lived experiences of people living with FXS all over the world from 1980 to 2019. Hammarberg et al. (16) reported that qualitative methods should be used to answer questions regarding experiences, meaning, and perspective from the participant's angle. Additionally, Seers (17) noted that findings from primary qualitative research could contribute, through a qualitative synthesis, to a greater understanding of a research area. Hence only qualitative articles will be included in our study.

### Identification of Research Question

To guide the search strategy and ensure that a broad range of literature was captured, we asked “what are the lived experiences of people living with FXS?” This question helped us capture the challenges, frustrations, and coping mechanisms that entail living with FXS.

### Searching for Relevant Studies

We identified articles by searching through four electronic databases; PubMed, Scopus, African Wide information and Web of science. The electronic search strategy was developed by the first author (KK) and an experienced librarian at the University of Cape Town. An example of the search query used is seen bellow (Table 1), where the original search was done in May 2019.

**Table 1:** Search strategy used in the four data bases (PubMed, Scopus, Web of science and African Wide Information).

**Search component**	**Search terms**
Caregivers	Caregiver OR brother OR sister OR mother OR father OR sibling OR parent OR spouse OR spouses OR carrier OR patient
Experiences	Concern OR stigma OR custom OR belief OR stressor OR culture OR challenge OR stress OR resilience OR psychology OR socioeconomic OR Religion OR coping OR behavior OR (mental suffering) OR Adapt OR emotional OR (Quality of life)
Fragile x syndrome	(Fragile X Syndrome) OR (Fragile X associated Tremor Ataxia Syndrome) OR (Fragile X associated Primary Ovarian Insufficiency) OR (Fragile X Mental Retardation Syndrome)
Qualitative research	(Qualitative study) OR (qualitative research) OR (empirical research)

### Selecting Studies and Charting the Data

The electronic search results were downloaded into EndNote X9.2 which is a bibliographic software. The first (KK) and fourth (SN) authors collaborated to remove duplicates and screened articles to determine whether they should be included in this review. Identified articles were then broadly coded by these two authors using NVivo 12 software. The data obtained was then grouped to form themes. We identified four overarching themes in the articles included in the analysis which are reported in a narrative format for this paper.

## Results

### Description of Identified Studies

**Figure 1:**
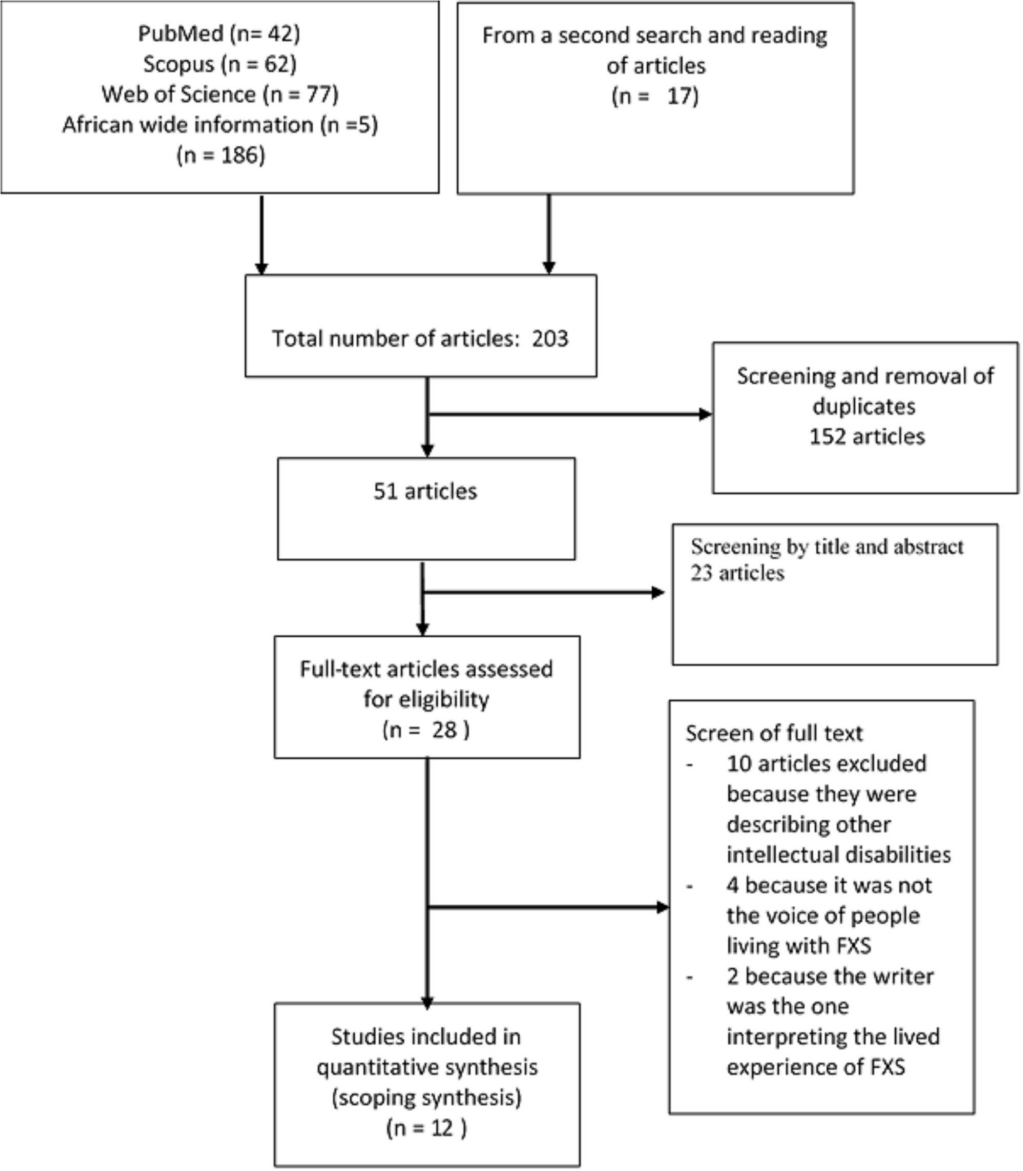
Chart describing the article selection process.

Overall, the search strategy identified 203 records; 186 from the electronic search results and 17 records from a second search and reading references of retrieved articles. After duplicates were removed, 51 records were screened. A total of 28 full-text articles of potential relevance were retrieved and screened; 16 articles were subsequently excluded. Twelve articles were retained for the final synthesis (Figure 1).

### Overview of Study Characteristics

The included studies were from English literature and captured the lived experiences of people directly involved in or affected by FXS. Most studies originated from North America (*n* = 9 for USA, *n* = 1 for Canada), one from the Netherlands and one from South Africa. All the studies involved standard qualitative methods (Interviews and Focus Group Discussions) with the parents of children with FXS, and the majority (n = 07) involving only mothers (Table 2).

**Table 2:** Demographic information of the identified studies.

**References**	**Title**	**Country**	**Participants**	**Method used**	**Site**	**Themes**
Brady et al. (19)	Communication in Young Children with Fragile X Syndrome: A Qualitative Study of Mothers' Perspectives	USA^a^	Mothers (*n* = 55)	Interviews	Home and university	Grief; Challenges; Copping strategies
Feinstein et al. (20)	“We Don't Have a Plan. We Should Be Working on a Plan.”: Obstacles to Caregiver Transition Planning for Individuals with Fragile X Syndrome	USA^a^	Caregiver (*n* = 37)	FGD^b^ and Interviews	Phone calls	Grief; Challenges; Copping strategies; worries about the future
Michie et al. (21)	Narrating Disability, Narrating Religious Practice: Reconciliation and Fragile X Syndrome	USA^a^	Mothers (*n* = 60)	Interviews	Home	Grief; Copping strategies
Minnes et al. (25)	Parent views on enhancing the quality of health care for their children with Fragile X Syndrome, autism or Down syndrome	Canada	Parents (*n* = 71)	FGD^b^	Conference room	Challenges
Muller et al. (28)	Mothers' perspectives on challenging behaviors in their children with Fragile X Syndrome	USA^a^	Mothers (*n* = 53)	Interviews	Home and university	Challenges
Poehlmann et al. (22)	Family Experiences Associated with a Child's Diagnosis of Fragile X or Down Syndrome: Evidence for Disruption and Resilience	USA^a^	Mothers (*n* = 11)	Interviews	Home	Grief; Challenges;
Reines et al. (30)	Parental Perspectives on Pharmacological Clinical Trials: a Qualitative Study in Down Syndrome and Fragile X Syndrome	USA^a^	Parents (*n* = 9)	Interviews	Phone calls	Worries about the future
Van Remmerden et al. (26)	Growing up with Fragile X Syndrome: Concerns and Care Needs of Young Adult Patients and Their Parents	Netherlands	Parents and patients (*n* = 38)	FGD^b^ and Interviews	Home and phone	Challenges;
Visootsak et al. (23)	Diagnosis of Fragile X Syndrome: A Qualitative Study of African American Families	USA^a^	Mothers (*n* = 10)	Interviews	Phone calls	Grief; Copping strategies
Weber et al. (24)	Understanding Fragile X Syndrome from a mother's perspective: Challenges and resilience	South Africa	Mothers (*n* = 1)	Interviews /journal/ field notes	Home	Grief; Challenges; Copping strategies
Weber et al. (29)	Voice of People with Fragile X Syndrome and Their Families: Reports from a Survey on Treatment Priorities	USA^a^	Patents (*n* = 8)	Interviews	Online	Challenges
Wheeler et al. (27)	Perceived Quality of Life in Mothers of Children with Fragile X Syndrome	USA^a^	Mothers (*n* = 10)	Interviews	Home	Challenges; Copping strategies

a *United States of America*.

b *Focus Group Discussion*.

### Grief and Response to Diagnosis

Grief is a response to the loss of someone or something cherished. When people grieve, they go through five stages which are denial, anger, blame, depression, and acceptance (18). Six of the studies included in this analysis describe grief as an important aspect of the lived experience of caring for a child with a FXS diagnosis (19–24). A quote from a participant in Visootsak et al.'s study describes this well: “I was devastated. I felt like I was grieving for a child that was still living” (23). Michie et al. also describe parents losing their sense of direction following the diagnosis (21).

Denial, the first stage of grief, was described by three authors (20–22). After receiving the diagnosis, parents go through a series of negative emotions dominated by denial. “Why would something be wrong with one of my kids?” (21). Feinstein et al. describe that denial does not only happen after receiving a diagnosis; it may also play a factor in delaying the search for a diagnosis; “*I didn't think about these things before we found ourselves in this situation*” (20). Similarly, Poehlmann et al. quoted some parents resolved to dismiss concerns about their children; “*In his first year, I remember my father-in-law asking if there was something on his eyes. I said, no. It's normal for kids to look kind of cross-eyed in their first years”* (22).

Other stages of grief described by the articles included in this analysis are anger and self-blame. One of the papers reported that parents were angry with God and considered their child's illness a punishment. Some parent attributed their child's illness to “bad karma” (21). Being angry with other people in the community was mainly a result of micro-aggression relating to FXS; “*When somebody says, “Joey's blessed to have you,” I'm like… “So, what in my life have I done that I deserve this much work?”* (21). *Self-blame was another common emotion described by three authors* (21, 22, 24). These authors narrate how receiving a FXS diagnosis generated panic attacks in mothers, relating to feelings that they caused their child's condition.

Most parents were frustrated with the long odyssey of obtaining the diagnosis of their child. The lack of knowledge of physicians and pediatricians with regards to FXS, was a concern for parents in this period. The atypical presentation of children may make FXS hard to diagnose, and the parents reported that their children received treatment for the child's symptoms before finally requesting chromosomal or FXS testing (22–26). Poelhmann et al. narrated situations of distress arising from the difficulties of the child to attain developmental milestones or the long and difficult process of getting to a diagnosis. After obtaining the diagnosis, mothers were left with limited options and did not know what to do next.

“*We were just blown out of the water with no clue… it changed our entire life about everything, even who we are… I have in my mind a picture of just walking into doors. Total confusion… it was almost like it had a feeling of throwing you out in the sea and saying I hope you can swim”* (22).

However, parents who manage to accept this condition keep in mind that they will play the role of primary care provider for the rest of their lives (19). Although mothers report receiving some support from their physicians (24), they disclosed that these practitioners lack empathy in giving them the result of their children. Poehlmann et al. recounted a scenario where a physician was not sensitive in giving back a FXS result to a parent;

“*We hadn't heard for over a month, the diagnosis… I called him and the doctor came to the phone and said, “Was I supposed to give you the diagnosis?” And I said, “Yes”… and he said “Well then… your child tested positive for Fragile X.” He didn't ask if anybody was there with me or if there was a time when my husband and I could come in… that is definitely a poor way to tell you that”* (22).

Upon receiving the diagnosis, parents described receiving support from spouse and extended family members who could listen and empathize with them (21, 23, 24, 27). Wheeler et al. recounted the life of a mother who described how having a supportive husband and/or extended family help to improve her quality of life;

“*They (husband's family) have accepted the boys and include the boys and me… And I feel like I can go to them. If we're at a family function and the boys are acting wild, they're like “Oh, so what? They do what they do.” Everyone's supportive and understanding and helpful”* (27).

Also having a role model was a great support. Weber (2016) stated the role a psychologist with special needs played in the life of a caregiver in an African setting;

“*I know the lady I told you that passed away, remember, she was very good, she was very good… because she also had a disability. And looking at her made me appreciate life and made me realize that if she can carry on each day, so can I*” (24).

### Challenges of Caring for a Child With FXS

Ten authors described the challenges of caring for a child with FXS (19, 20, 22–29). Throughout childhood and adolescence, parents described challenges in dealing with behavioral difficulties, especially when skills like speaking and communication are compromised. Parents also described concerns about their children's ability to live independently. More so, academic performance, self-care and behavior were the main concern for parents taking care of male FXS patients while social issues were the main concern for parents of females FXS patients (29). Worries about the future and dealing with behavioral challenges ultimately reduced the quality of life of families.

Concerning behavioral challenges, stubbornness, stereotypic behaviors, aggression, self-injury, impulsivity and social deviance were some behaviors recounted by parents (20, 24, 26, 28). These behaviors were thought to be a result of the non-verbal expression of the children or a way to escape demands, seek attention or obtain tangibles (24, 26, 28). Socially, children were reported to behave inappropriately because they are unable to respect the private space of others and are also unable to change conversation tone or topics. This keeps mothers frustrated and embarrassed (28). Moreover, food control, keeping a good personal hygiene and sleep disorders were worries for parents and caregivers (20, 26, 28).

In addition to social and behavioral challenges, communication patterns of children living with FXS was a worry for care givers (19, 20). Parents believe that their children are not always understood because they communicate non-verbally. Mothers reported frustration with their inability to understand what their child wanted to say. Brandy et al. elaborated on different communication challenges and strategies adopted by mothers. Most mothers try to guess what their child was trying to say, and they resolve to adopt a trial and error strategy which is frustrating for the mother and child.

“*I just try a couple of different things and I either hit the right one or I distract him or he gets interested in something else and he kinds of forgets, which is kind of sad, you know, if the little guy isn't getting what he wants”* (19).

Supervisory experiences were also addressed by caregivers as challenging. Most children with FXS cannot be on their own without supervision and even adult persons with mild forms of intellectual disability still need some coaching and monitoring (20, 26). Van Remmerden et al. illustrated this dependency by recounting the life of a mother who had a daughter with FXS;

“*My daughter lives on her own but she is not independent. My husband and I do everything for her: the finances, cooking and cleaning. Sometimes I feel like I let a 14-year-old move out of the house”* (26).

Parents described situations of having difficulties obtaining specialized services or support from the community. In the Dutch (26) and African (24) studies, parents described limited knowledge of health care workers of FXS and limited specialist care and support facilities available. Van Remmerden et al. elaborated this thought from a mother; “*As a parent, you always need to explain what FXS is, even to some physicians. It's so typical for FXS. When you say my daughter has Down syndrome, everybody knows what you're talking about”* *(**26**)*. Parents in a study from the US reported that even though there is some form of specialized support for FXS in that country, the waiting lists are long, and parents receive little information about how to care for their child following diagnosis (25). A lack of support from family members especially the spouse can constitute a source of distress (22–24). Poehlmann et al. quoted a family who did not have enough support from their relatives and the caring duty was relegated to the mother;

*“He was fussy, and I had three kids and was working full-time, and my husband was working and gone most of the time. So, I had to take care of them all at night, and it was a very difficult time. My parents were in a town 40 miles away and they were older, so they really couldn't help much. And my husband's folks were in town, but they weren't much of a support. They had a hard time handling our son's behaviors and outbursts and so they weren't around a lot because they couldn't understand him. So, we really didn't have a lot of support”* (22).

Weber (2016) reported that the rarity of FXS in that country also impacts on one patient's ability to find support through more formal means;

“*It is not easy for someone to go through that. You need support… even with the group we had, there was no kids there that had fragile X. It was just my kids.… The other support groups they were of different disabilities”* (24).

Furthermore, two authors described challenges with the transition from childhood to adulthood (20, 26). Difficulties obtaining services in adulthood was another concern because the available services are limited. In addition, caregivers worry about the complex bureaucratic work which they must go through in order to meet the vocational, residential and caregiving needs of their children. This complex process motivated mothers to give up their personal ambitions and become the primary caregiver for their children.

Moreover, challenges with family planning, romantic life and sexual deviance were addressed by one author. Female FXS patients were concerned about having children but were discouraged when they found out the stress associated with taking care of a FXS child through their parents. Hence, parents doubted their ability to raise a child and much preferred their FXS children not to attempt to have their own (26).

Finally, starting and maintaining a romantic relationship for an individual affected with FXS is very difficult (26). Sexual deviance was reported by some parents and it was characterized by exhibiting their private parts or masturbating in public places. This behavior left parents worried since their children could be abused or assaulted by others who do not know their FXS status.

### Coping Strategies

Fragile X Syndrome is a disabling condition both for individuals and families and from this review, it appears as if there are two dominant coping strategies employed by the families affected by FXS. Some become advocates of FXS whilst others give up their dreams and concentrate on the care of their children. To become an advocate of FXS, caregivers describe taking courses that empower them to speak about the illness and to obtain support for their care (19, 20, 23).

Obtaining support was another way to cope when parents had children with FXS. Previously, we described that some of the FXS parents end up feeling isolated and depressed—but a subset of parents seems to have found more positive ways of dealing with FXS. What seems to make the difference is those parents' ability to seek support, either from external support services or from faith. Wheeler et al. described the persevering and positive attitude of a mother in seeing the progress made by her kids;

“*I know all those services out there. I'm very positive. I know I'm not missing out on anything. I know I'm on top of the game with my kids. I keep busy. I have a social life. I have friends”* (27).

Besides, some caregivers rely on their faith in God. Reconciliation is the term used by Michie et al. to represent the transition from viewing FXS as a burden or challenge to being more a blessing or God's will (21). What seems to be different here is that these parents seem to have developed a high level of self-esteem and positive outlook (19–21, 23, 24, 27).

### Worries About the Future

Out of the 12 articles retrieved, three describe worries about the future which included the need to plan for housing, financial security and caregiving (20, 24, 30). Feinstein et al. described instances with parents not having a well-structured plan. In this case, parents were so busy with their daily activities that they did not feel the need to plan for the future of their children;

“*When [my son] was younger, I thought, yeah, I was 30, 35— that's a good age if they must start living on their own and moving in there. Well, golly geez, that's 4–9 years away. It's time to get moving”* (20).

On the other hand, those with a partial/concrete plan based their plans on their families or organization they created. They hoped that these entities will provide housing or financial aids to their offspring when they will not be around;

“*Right now, I think that if something happened tomorrow, my parents are still alive. I've got sisters and a brother. My husband has a sister, and we have close friends. They all understand [daughter living with FXS]'s situation. I think someone would step up. I would hope that my son as he got older would take on the responsibility himself”* (20).

Moreover, participation in clinical trials for targeted therapies in the future was a concern for caregivers. Reines et al. describe the willingness of parents to participate in experiments that targeted the disease and not the symptoms. They think participating in the research will either improve their child's condition or give hope for the discovery of a treatment in the future which will help other families suffering from the same condition (30). However, these parents were worried about long term and transient side effects of the medication and the logistics that comes with the clinical trial. Also, taking the decision to participate in a trial is a big responsibility which parents are ready to share with family members (spouse, grandparents, the child), health care workers, and other parents in the FXS community.

## Discussion

**Figure 2:**
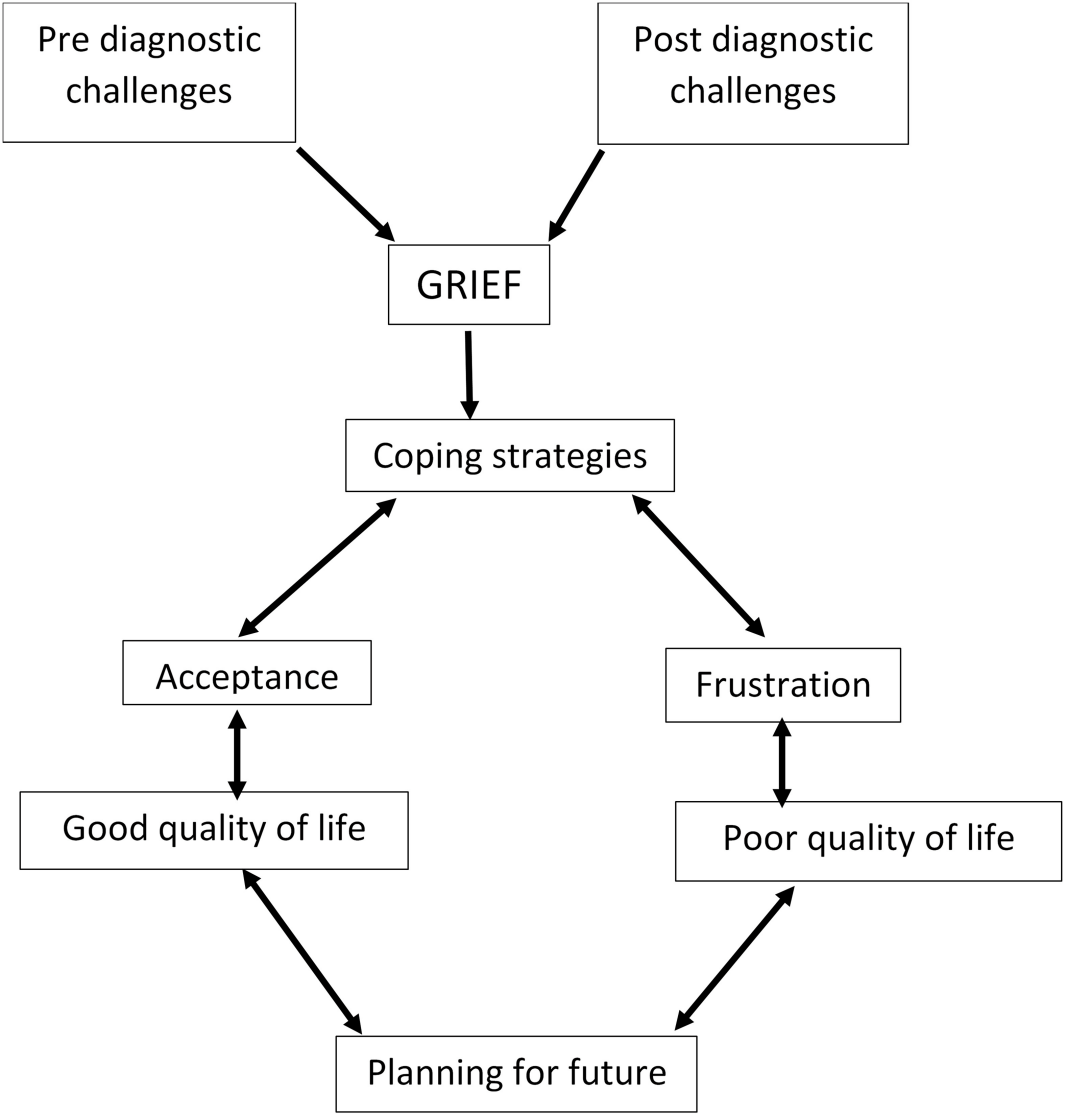
Flow chart describing the lived experiences of families with FXS.

This scoping review is the first to summarize the qualitative literature on lived experiences of FXS families. Our results provide a comprehensive overview of the challenges, grief experiences and coping strategies developed. The lived experiences with FXS could be grouped into the pre-diagnostic and the post-diagnostic period. Before the diagnosis, several parents of FXS children struggled to receive a diagnosis for their children. This is a time-consuming process and parent can find themselves spending money which could lead to impoverishment (31, 32). Grief reactions can extend from the pre-diagnostic period to the post-diagnostic period. Parents who accept the condition understand that they will be the primary caregivers of their children and could then integrate that in their living style. They then start to plan for the future of their children (31). However, in some instances, parents are unable to accept their child's condition. They are those who finally have a poor quality of life (27). Moreover, challenges encountered when living with FXS can either be before or after the diagnosis. These challenges are related to the basic skills of their children and their ability to integrate in society due to communication and behavioral difficulties. Coping strategies that were implemented were related to family and community support (Figure 2).

### Practice Implications

Most studies illustrate the importance of patients' experiences with the health care system. Adults described the need for a good relationship with their health care providers, to be treated with respect and as a whole person, a need for understandable information about the diagnosis, and valued health professionals who could assist whenever needed. York et al. reported that educators and health caregivers taking care of down syndrome patients, autistic children and FXS children demonstrated to have limited knowledge of FXS (33). Results from the present review of the literature, in general, show that lack of expertise among health care providers is a major barrier for people with FXS (23, 25). This lack of knowledge about FXS can result in important medical consequences. This may include delays in obtaining an accurate diagnosis, psychological stress due to inappropriate responses to delivering the diagnosis and providing support to affected individuals. In contrast, from the apparent unwillingness from health care providers to get involved or to seek information about FXS created a lack of trust.

### Research Recommendations

The experiences of living with FXS has never been reviewed before. The present scoping review examined qualitative literature which described the lived experiences of FXS through the narratives of caregivers. From our findings, we could identify gaps in two areas which are: a lack of FXS qualitative studies in Africa, and the limited knowledge of FXS by health care workers. In Africa, very limited literature is found concerning rare monogenic diseases like FXS with only one study found (24). Lim et al. reported that the burden and caring responsibilities of children suffering from rare neurological disorders in china typically falls on the parents and this is the case with FXS (34). Furthermore, Von de Lip et al. conducted a qualitative systematic review on rare diseases and found that most studies were from Europe and America (35). Therefore, there is an urgent need to research on such knowledge for FXS in most part of the World, and particularly in Africa.

### Study Limitations

There are some limitations associated with the conduct of this scoping review that need to be acknowledged. The first was the search strategy which focused on English language studies. However, there could be other relevant literature which was published in other languages. Most of the studies reviewed had mother's voices more prominent—despite the title of the papers invariably using the word “parent or caregivers.” Less is known about what fathers are saying, and we cannot assume that the lived experiences are homogenous. Other limitations are related to the heterogeneity and variable scientific quality of the 12 papers included in the review, as well as the focus on qualitative approaches in our design. Indeed, quantitative data obtained from standardized scales measuring quality of life and living experiences in parents with FXS could have provided additional angle and insights.

## Conclusion

Individuals caring for children suffering from FXS face challenges beyond medical issues. Many of the challenges could be diminished by more education and creating awareness about FXS and other inheritable diseases in families and communities. The findings highlight the need for more research on the lived experiences of families with FXS on the African continent while exploring the experiences of fathers and younger individuals to complete the picture provided by this review on caregivers.

## Author Contributions

KK and SM literature search, interpretation, and writing. SN, JD, and AW design, interpretation, and writing.

### Conflict of Interest

The authors declare that the research was conducted in the absence of any commercial or financial relationships that could be construed as a potential conflict of interest.
